# Review on the protective mechanism of astragaloside IV against cardiovascular diseases

**DOI:** 10.3389/fphar.2023.1187910

**Published:** 2023-05-11

**Authors:** Chunkun Yang, Qingquan Pan, Kui Ji, Zhuang Tian, Hongyuan Zhou, Shuanghong Li, Chuanchao Luo, Jun Li

**Affiliations:** ^1^ Guang’anmen Hospital, China Academy of Chinese Medical Sciences, Beijing, China; ^2^ Department of Emergency, Weifang Hospital of Traditional Chinese Medicine, Weifang, China

**Keywords:** astragaloside IV, anti-oxidative stress, apoptosis, fibrosis, inflammation, energy metabolism, cardiovascular diseases

## Abstract

Cardiovascular disease is a global health problem. Astragaloside IV (AS-IV) is a saponin compound extracted from the roots of the Chinese herb *Astragalus*. Over the past few decades, AS-IV has been shown to possess various pharmacological properties. It can protect the myocardium through antioxidative stress, anti-inflammatory effects, regulation of calcium homeostasis, improvement of myocardial energy metabolism, anti-apoptosis, anti-cardiomyocyte hypertrophy, anti-myocardial fibrosis, regulation of myocardial autophagy, and improvement of myocardial microcirculation. AS-IV exerts protective effects on blood vessels. For example, it can protect vascular endothelial cells through antioxidative stress and anti-inflammatory pathways, relax blood vessels, stabilize atherosclerotic plaques, and inhibit the proliferation and migration of vascular smooth muscle cells. Thus, the bioavailability of AS-IV is low. Toxicology indicates that AS-IV is safe, but should be used cautiously in pregnant women. In this paper, we review the mechanisms of AS-IV prevention and treatment of cardiovascular diseases in recent years to provide a reference for future research and drug development.

## 1 Introduction

Cardiovascular diseases (CVD) are a global health problem, causing the largest number of deaths worldwide, and their incidence is still increasing. It is estimated that approximately 20 million people died from CVDs in 2016, accounting for 31% of all deaths worldwide (Tan et al., 2020). There is no doubt that CVD has become a public health crisis. Tu Youyou’s discovery of artemisinin shows that Chinese herbal medicine is a great treasure. Over the past few decades, natural compounds derived from Chinese herbs have become important resources for drug research and development, particularly for treating CVD (Lin et al., 2022).

*Astragalus membranaceus* (known as Huangqi in China; HQ) is a plant of the genus *Astragalus* in the leguminous family, whose dried roots are used in traditional Chinese herbal medicine. HQ is one of the most widely used traditional Chinese medicines and was first described in *Shennong’s Classic of Material Medical*. It has the effect of tonifying qi and uplifting Yang, solidifying the surface, and acting as an antiperspirant, collecting sores, and generating muscles. Clinically, a variety of Chinese patent medicines and injections are made from or contain HQ, including *Astragalus* injection, *Astragalus polysaccharide* injection, *Yupingfeng* Powder, *Xuefu Zhuyu* Decoction, *Huangqi Siwu* Decoction, etc (Fu et al., 2011; Lu et al., 2011).

**FIGURE 1:**
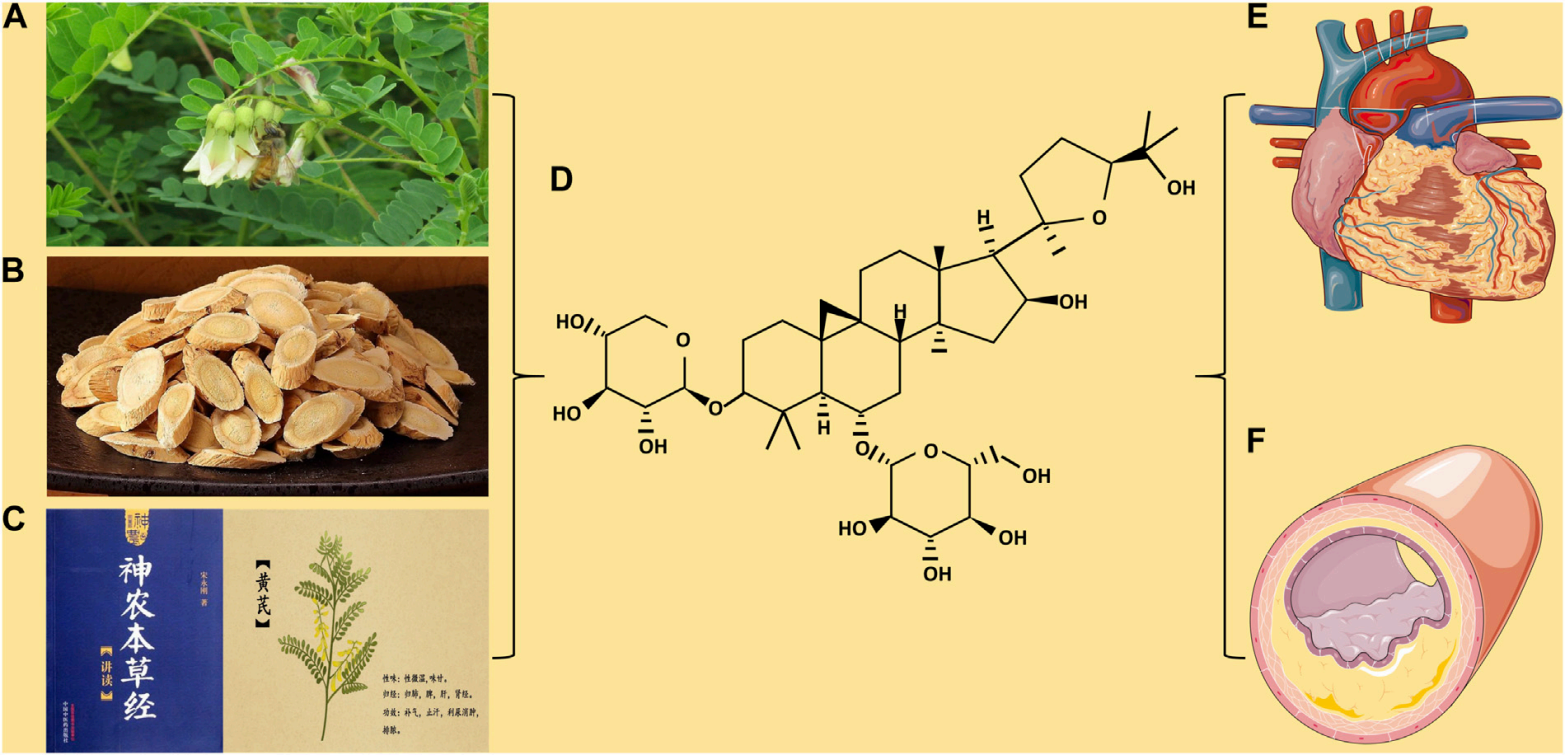
**(A)** *Astragalus membranaceus*. **(B)** *Astragalus membranaceus*. Decoction pieces. **(C)** *Shennong’s Classic of Material Medical* and picture of *Astragalus membranaceus*. **(D)** Chemical structure of Astragaloside IV. **(E)** The protection of Astragaloside IV on myocardial disease. **(F)** The protection of Astragaloside IV on blood vessels.

Astragaloside IV (AS-IV) is a purified small-molecule saponin that is one of the main active components of astragaloside (Ren et al., 2013). Previous studies have found that AS-IV has various activities, such as anti-oxidative stress (Ko et al., 2005), anti-inflammatory (Zhang and Frei, 2015), anti-apoptosis (Huang et al., 2012), calcium balance (Lu et al., 2018), diabetes (Lv et al., 2010) and anti-CVD (Han et al., 2011). Currently, studies on the AS-IV are limited. To further determine the protective mechanism of AS-IV against CVD, we reviewed recent research reports on AS-IV and provided a systematic summary (Figure 1).

## 2 Cardiac protection

### 2.1 Alleviating oxidative stress

Myocardial Ischemia/reperfusion (I/R) injury is a significant clinical problem because of the significant explosion of free radicals during the first few minutes of I/R due to the reintroduction of oxygen, which, coupled with reduced antioxidant activity, makes the heart muscle extremely vulnerable (Moens et al., 2005). Energy metabolism provides substrates for reactive oxygen species (ROS) and regulating the utilization of myocardial metabolic substrates is a therapeutic strategy for mitigating ROS-related injuries. Succinic acid is an important metabolite of the tricarboxylic acid (TCA) cycle. It has previously been reported that succinic acid accumulates during myocardial ischemia and can be consumed in large quantities during reperfusion to produce ROS (Chouchani et al., 2014). Jiang et al. found that AS-IV (40 mg/kg) prevented the accumulation of succinic acid in the myocardium of Sprague Dawley (SD) rats after I/R, thus reducing the production of ROS. In addition, AS-IV activates the Nrf2/HO-1 signaling pathway and upregulates the expression of antioxidant enzymes, thereby protecting cardiomyocytes from I/R damage (Jiang et al., 2019). The mitochondrial permeability transition pore (mPTP) plays an important role in the pathogenesis of myocardial I/R injury (Bauer and Murphy, 2020). Glycogen synthase kinase 3β (GSK-3β) is a mature component that promotes apoptosis (Frame and Cohen, 2001). He et al. found that AS-IV (50 μM) phosphorylated GSK-3β through NO/cGMP/PKG pathway, thereby inhibiting oxidative stress-induced mPTP opening of H9C2 cells (He et al., 2012). In another study, AS-IV (5 and 10 mg/kg) alleviated myocardial I/R damage in SD rats by modulating the PI3K/Akt/GSK-3β signaling pathway (Wei et al., 2019).

**FIGURE 2:**
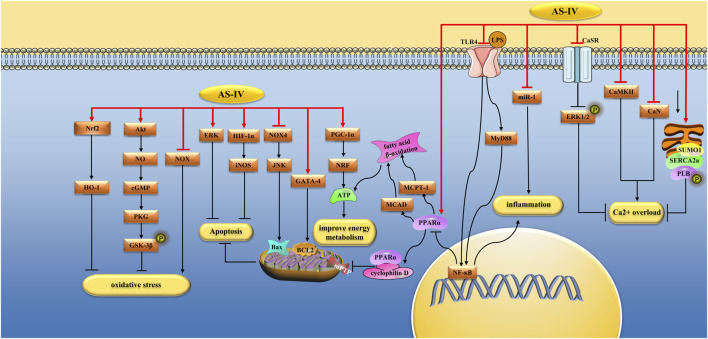
Pharmacological effects of AS-IV on myocardium. AS-IV protects myocardium by anti-oxidative stress, anti-inflammatory, regulating calcium balance, improving energy metabolism, and anti-apoptosis pathways.

Doxorubicin (DOX) is a classic antitumor chemotherapy drug that induces fatal cardiotoxicity. DOX causes oxidative stress by increasing the expression of NADPH oxidase 2 (NOX2) and NOX4 in rat hearts, leading to cardiomyopathy (McLaughlin et al., 2017). Lin et al. found that AS-IV (40 mg/kg) significantly reduced myocardial injury, myocardial cell apoptosis, cardiac fibrosis, and cardiac dysfunction in DOX-treated C57BL/6 mice by inhibiting the expression of NOX2 and NOX4 (Lin et al., 2019). Histone deacetylases (HDAC) work with histone acetylases to regulate the homeostasis of acetylated histones *in vivo*. Zhang et al. found that AS-IV (12.5 and 50 µM) could inhibit HDAC activity, thereby protecting HL-1 mouse cardiomyocytes from oxidized low-density lipoprotein (ox-LDL) oxidative damage (Zhang et al., 2021) (Figure 2).

### 2.2 Anti-inflammatory

Sepsis is a systemic inflammatory response that occurs after infection. Sepsis-induced cardiovascular dysfunction is the leading cause of death in critically ill patients (Bone et al., 1992). Lipopolysaccharide (LPS) produced by bacterial endotoxins is considered the main factor leading to cardiac dysfunction in sepsis, and its mechanism may be related to the release of many inflammatory cytokines (Zanotti-Cavazzoni and Hollenberg, 2009). Toll-like receptor 4 (TLR4) is a major LPS receptor and a key mediator of pro-inflammatory responses. Zhang et al. found that AS-IV (20, 40, and 80 mg/kg) significantly improved cardiac function, myocardial cell viability, and pathological changes in lipopolysaccharide-induced C57BL/6J mice. It also reduces the level of interleukin-1β (IL-1β), IL-6, and tumor necrosis factor α (TNF-α) in myocardial tissue. In addition, AS-IV increased the ratio of adenosine triphosphate (ATP)/adenosine monophosphate (AMP) in myocardial tissue and promoted the expression of ATP synthase. Its mechanism was related to the inhibition of the TLR4/NF-κB pathway and upregulation of proliferator-activated receptor α(PPARα) (Zhang et al., 2019a). Zhao et al. found that AS-IV (20 mg/kg) significantly reduced cardiac dysfunction and inflammatory mediator production in LPS-induced male C57BL/6 mice. This mechanism is related to the inhibition of NF-κB and the activation of the PI3K/Akt signaling pathway (Zhao et al., 2015a). Su et al. evaluated the effect of AS-IV on cardiac dysfunction of SD rats induced by cecal ligation and puncture (CLP). They found that AS-IV (30 mg/kg/d) improved the survival rate of rats after CLP surgery, improved cardiac function, and inhibited the secretion of pro-inflammatory cytokines. *In vitro* studies, AS-IV (100 μmol/mL) mitigated LPS (40 μg/mL) damage to H9C2 cells. This mechanism is related to the inhibition of the NOX4/JNK/Bax signaling pathway (Su et al., 2022). Yang et al. injected isoproterenol (5 mg/kg/d) peritoneally into SD rats to make myocardial hypertrophy models, and they found that AS-IV (20,40 and 80 mg/kg) inhibited myocardial hypertrophy and serum levels of TNF-α and IL-6, the mechanism of which was related to inhibition of TLR4/NF-κB signaling pathway (Yang et al., 2013). Shi et al. found that AS-IV (80 mg/kg/day) reduced myocardial inflammation in SD rats with acute myocardial infarction (AMI) by inhibiting the TLR4/MyD88/NF-κB pathway (Shi et al., 2021). Huang et al. found that AS-IV (40 mg/kg/d) reduced myocardial cell apoptosis in SD rats after CLP operation and reduced serum inflammatory cytokines IL-6, IL-10, and high mobility group protein B1 (HMGB-1), the mechanism of which is related to the inhibition of IKK/NF-κB pathway (Huang et al., 2021a).

MicroRNAs (MiRNAs) regulate gene expression after transcription. miR-1 has been identified as a muscle-specific miRNA that is upregulated in rats with myocardial infarction (MI) (Shan et al., 2009). Wang et al. found that AS-IV (80 mg/kg) alleviated LPS (10 mg/kg) induced cardiac dysfunction in SD rats by inhibiting miR-1-mediated inflammation and autophagy. *In vitro*, AS-IV (10 g/mL) alleviated LPS-induced H9C2 cell damage (10 g/mL) *via* the same mechanism (Wang et al., 2022). In another study by the same team, AS-IV (80 mg/kg) alleviated LPS-induced cardiac dysfunction in SD rats by inhibiting miR-1-mediated apoptosis. Mitochondrial energy metabolism and calcium signaling pathways are also involved in cardiac insufficiency. In this study, AS-IV (80 mg/kg) decreased calcium/calmodulin-dependent protein kinase II (CaMKII) expression, improved ryanodine receptor 2 (RyR2) and sarcoplasmic reticulum Ca^2+^-ATPase2a (SERCA2a) protein expression to inhibit myocardial cell damage caused by calcium overload. Peroxisome proliferator-activated receptor-gamma coactivator-1α (PGC-1α), and mitochondrial transcription factor A were involved in mitochondrial energy metabolism, and AS-IV (80 mg/kg) increased the expression of these two types of proteins (Wang et al., 2021a) (Figure 2).

### 2.3 Regulating calcium balance

Dysregulation of intracellular calcium homeostasis plays an important role in myocardial cell injury induced by I/R or hypoxia/reoxygenation (H/R). An excessive increase in intracellular Ca^2+^ concentration leads to Ca^2+^ overload, leading to myocardial cell injury (Zucchi et al., 2001). The calcium-sensing receptor (CaSR) is a member of the G protein-coupled receptor (GPCRs) superfamily involved in systemic calcium homeostasis. Overactivation of CaSR increases intracellular Ca^2+^ levels, thereby inducing cardiomyocyte apoptosis (Lu et al., 2013). Yin et al. (Yin et al., 2019) found that AS-IV (80 mg/kg/day) significantly reduced MI size, serum cardiac troponin (cTnI) levels, and myocardial cell apoptosis in SD rats with myocardial I/R injury. *In vitro*, AS-IV (60 mol/L) pretreatment significantly increased cardiomyocyte viability, decreased lactate dehydrogenase (LDH) release and intracellular Ca^2+^ levels, and alleviated cardiomyocyte apoptosis. This mechanism is related to the downregulation of CaSR expression in cardiomyocytes and the upregulation of extracellular signal-regulated kinases1/2 (ERK1/2) phosphorylation (Yin et al., 2019). Lu et al. found that AS-IV treatment (80 mg/kg/day) inhibited the activation of CaSR, CaMKII, and Ca^2+^/Calcineurin (CaN) in the myocardium of isoproterenol-induced SD rats and inhibited the nuclear translocation of NFAT3, thereby alleviating myocardial hyperplasia and cardiomyocyte apoptosis (Lu et al., 2018). SERCA2a is another important regulatory protein that is responsible for Ca^2+^ homeostasis in cardiomyocytes. Xu et al. found that AS-IV (30 mol/L) inhibited H/R-induced Ca^2+^ overload and prevented the reduction in SERCA2a enzyme activity and SERCA2a mRNA and protein levels (Xu et al., 2008). Phospholamban (PLB), a small protein in the sarcoplasmic reticulum (SR), is a key regulator of SERCA2a. The phosphorylation of PLB enhances SERCA2a function by increasing its Ca^2+^ affinity (Koss and Kranias, 1996). In another study, Xu et al. found that AS-IV (5 and 10 mg/kg/day) reduced isoproterenol-induced myocardial injury in SD rats through a mechanism related to increased phosphorylation of PLB and SERCA2a protein expression (Xu et al., 2007). Decreased expression and activity of SERCA2a protein can lead to impaired calcium homeostasis in cardiomyocytes. Mitochondria-sourced ATP drives SERCA2a-mediated Ca^2+^ uptake into the SR. In addition, activation of small ubiquitin-like modifier1 (SUMO1) enhanced the binding affinity of SERCA2a to ATP. Dong et al. found that AS-IV could promote the expression of SERCA2a and the activation of SUMO1 (Dong et al., 2017) (Figure 2).

### 2.4 Improve myocardial energy metabolism

During fetal life, cardiomyocytes obtain energy mainly through glycolysis, which is converted to fatty acid β-oxidation after birth. However, in patients with heart failure (HF), the primary energy source for cardiomyocytes changes from fatty acid β-oxidation to glycolysis. Fatty acid oxidation provides more energy than glucose, and restoring cardiomyocytes to fatty acid use may be an effective treatment for HF. It has been shown that the expression of PPARα can stimulate the β-oxidation of fatty acids (Minnich et al., 2001). Dong et al. (Dong et al., 2017) found that AS-IV (0.3 and 1 mg/kg/d) can improve systolic myocardial function in mice with HF induced by transverse aortic contraction (TAC), and the mechanism is related to the activation of PPARα. *In vitro* studies, AS-IV not only inhibited the expression of hypertrophic marker genes in hypertrophic cardiomyocytes but also significantly inhibited the reduction of ATP, the mechanism of which is related to the inhibition of glycolysis and the acceleration of fatty acid β oxidation. Several studies have shown that mitochondrial Ca^2+^ uptake promotes ATP production to a certain extent (Di Benedetto et al., 2013). Cyclophilin D is a major regulator for the formation of mPTP. PPARα is involved in the opening of the mPTP by binding to cyclophilin D. Dong et al. (Dong et al., 2017) AS-IV could inhibit the significant decrease in the interaction between PPARα and cyclophilin D in the TAC group and the decrease in Ca^2+^ accumulation in the mitochondrial matrix.

PGC-1α is responsible for the metabolic transition from fatty acid oxidation to glucose oxidation. In addition, PGC-1α also regulates the expression and activity of ATP synthase subunit ATP5D, thus regulating energy biosynthesis. Zhang et al. found that AS-IV (80 mg/kg/day) had a protective effect against isoproterenol-induced myocardial hypertrophy in SD rats, increased the ATP/AMP ratio, reduced free fatty acid (FFA) content, and increased ATP5D expression. *In vitro* studies, AS-IV (100 μM) has the same protective effect on neonatal rat ventricular myocytes (NRVM) treated with isoproterenol, and the mechanism is related to the regulation of the NF-κB/PGC-1α signaling pathway (Zhang et al., 2015). Medium-chain acyl-CoA dehydrogenase (MCAD) and muscle carnitine palmitoyl transferase −1 (MCPT-1) are key enzymes involved in FFA oxidation, and their expression is controlled by PPARα (Sankaralingam and Lopaschuk, 2015). Tang et al. found that AS-IV (30 and 60 mg/kg/d) improved the cardiac function and structure, increased the expression of PPARα, MCAD, and MCPT1, and improved the utilization of FFA in rats with HF induced by abdominal aortic contraction (AAC) (Tang et al., 2018). The pathogenesis of diabetic cardiomyopathy is closely associated with metabolic disorders. PGC-1α stimulates the nuclear transcription factor nuclear respiratory factor (NRF), regulates cellular energy metabolism, and provides ATP to cardiac tissue (Koh et al., 2017). Zhang et al. induced diabetes in SD rats with streptozotocin (STZ) and found that AS-IV (10, 20, and 40 mg/kg/d) regulated energy metabolism by up-regulating PGC-1α and NRF, saving the abnormal energy metabolism caused by diabetes and thereby alleviating myocardial damage (Zhang et al., 2019b). In another study, Tu et al. found that AS-IV (10 mg/kg) mitigated I/R-induced cardiac insufficiency in SD rats and inhibited the reduction in ATP/AMP and ATP 5D proteins (Tu et al., 2013) (Figure 2).

### 2.5 Anti-apoptosis

Myocardial cell apoptosis is closely associated with HF. Studies have found that Patients with MI exhibit a certain degree of cardiomyocyte apoptosis (Saraste et al., 1997). The mitogen-activated protein kinase (MAPK) family comprises three kinases: c-Jun NH2-terminal kinases (JNKs), ERKs, and p38 enzymes (p38 MAPKs). The MAPK signaling pathway is involved in apoptosis. For example, inhibition of p38 and JNK can prevent myocardial cell apoptosis (Mackay and Mochly-Rosen, 1999; Mizukami et al., 2001). Sun et al. found that AS-IV (10 or 50 ng/mL) prevented H9C2 cell apoptosis induced by high glucose, fat (HG/HF), and hypoxia. Its mechanism is related to inhibiting the activation of the JNK and p38 signaling pathways and promoting the activation of the EKR signaling pathways. In animal experiments, AS-IV (10 and 50 mg/kg/day) protected the cardiac function of STZ-induced C57BL/6 mice and inhibited myocardial fibrosis and inflammation by regulating the MAPK signaling pathway (Sun et al., 2021a). Calpain-1 is a subtype of the cysteine protease family with Ca^2+^ and phospholipid-binding domains. When the intracellular free Ca^2+^ concentration reaches a trigger point, free Ca^2+^ binds to the Ca^2+^-binding domain, truncates downstream proteins, and induces associated reactions, including apoptosis (Momeni, 2011). Previous studies have shown that calpain-1 promotes apoptosis during myocardial I/R and pressure/overload (Suryakumar et al., 2010; Chen et al., 2011a). Mei et al. found that AS-IV (80 mg/kg/day) reduced isoproterenol-induced apoptosis of hypertrophic cardiomyocytes in SD rats by inhibiting calpain-1 and antioxidant stress (Mei et al., 2015). The transcription factor GATA-4 regulates the survival and adaptation response of cardiomyocytes under stress conditions, and the knockout of GATA-4 leads to cardiomyocyte apoptosis (Oka et al., 2006). Yang et al. found that AS-IV (0.5–300 μg/mL) could promote the expression of Bcl-2 by stimulating the overexpression of GATA-4, thereby reducing H/R injury-induced apoptosis in H9c2 cells (Yang et al., 2020). Hypoxia-inducible factor-1α (HIF-1α) is a key regulator of molecular hypoxia response, which can increase oxygen delivery or promote metabolic adaptation to hypoxia by activating gene transcription related to energy metabolism, angiogenesis, and apoptosis (Semenza, 2012). Si et al. found that AS-IV (50 μM) increased the strength of rat neonatal cardiac myocytes (RNCM) after ischemia and decreased the apoptotic index and LDH release by up-regulating HIF-lα/iNOS signaling pathway. In addition, AS-IV (20 μM) post-ischemic therapy protects the cardiac function of isolated I/R-injured hearts by reducing the infarct area, apoptotic index, and LDH release (Si et al., 2014) (Figure 2).

### 2.6 Anti-hypertrophy

**FIGURE 3:**
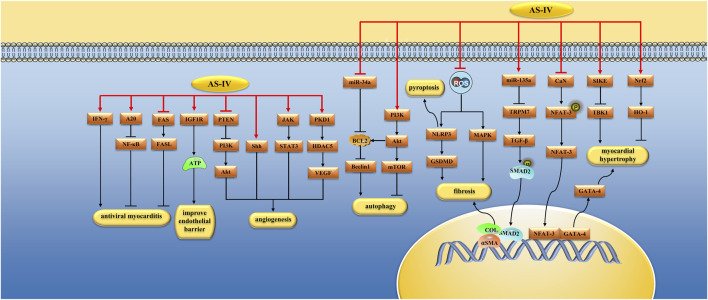
Pharmacological effects of AS-IV on myocardium. AS-IV protects myocardium by anti-cardiomyocyte hypertrophy, anti-fibrosis, regulation of autophagy, improvement of microcirculation, antiviral myocarditis.

Cardiac hypertrophy is an adaptive response of the heart against cardiac overloading, but continuous cardiac hypertrophy could accelerate cardiac remodeling and lead to HF in the future (Higashikuni et al., 2013). The suppressor of IKKϵ(SIKE) is a kind of ΙKKϵ and TANK-binding kinase 1 (TBK1) inhibiting factor. TBK1, also known as NF-κB activated kinase, promotes the translocation of NF-κB and causes inflammation (Shostak and Chariot, 2015). Liu et al. found that AS-IV (10 and 20 mg/kg/day) inhibited myocardial hypertrophy, inflammatory response, and myocardial cell apoptosis induced by aortic banding in C57BL6 mice, and the mechanism was related to the upregulation of SIKE and the inhibition of TBK1/PI3K/Akt activity. *In vitro* studies, AS-IV (50 μM) alleviated cardiomyocyte hypertrophy induced by angiotensin II (Ang II) by inhibiting these pathways (Liu et al., 2018). CaN is a serine/threonine protein phosphatase activated by calcium ions. Activated CaN binds to NFAT-3 transcription factors in the cytoplasm and dephosphorylates them. Dephosphorylated NFAT-3 is transferred to the nucleus to further interact with GATA-4 transcription factors to form complexes that participate in the development of myocardial hypertrophy. LPS is an antigenic component of the cell walls of Gram-negative bacteria that can cause cardiac hypertrophy (Liu et al., 2008). Lu et al. found that AS-IV (16, 32, and 64 µM) could reduce LPS-induced myocardial hypertrophy, and its mechanism was related to the inhibition of the CaN/NFAT-3/GATA-4 signaling pathway (Lu et al., 2014). The transcription factor nuclear factor-erythroid 2-related factor 2 (Nrf2) is a key regulator of cellular antioxidant defense. Nie et al. found that AS-IV (40 and 80 mg/kg/day) inhibited the degree of myocardial hypertrophy induced by AAC in rats and that this mechanism was related to the upregulation of the Nrf2/HO-1 signaling pathway (Nie et al., 2019a) (Figure 3).

### 2.7 Antifibrosis

Under pathological conditions such as inflammation and oxidative stress, cardiac fibroblasts (CF) can differentiate into myofibroblasts, accompanied by excessive accumulation of extracellular matrix (ECM) (Kania et al., 2009). Myocardial fibrosis is an important pathological change during the progression of HF (Yu and Xu, 2015). Transient receptor potential melastatin 7 (TRPM7) is a key mediator of fibrosis. The transforming growth factor-β (TGF-β)/Smad signaling pathway is the main pathway leading to myocardial fibrosis (Friedman et al., 2013). Wei et al. established a myocardial fibrosis model by subcutaneously injecting isoproterenol into SD rats and found that AS-IV (10 mg/kg/d) inhibited the increase of fibrosis-related proteins such as α smooth muscle actin (α-SMA) and collagen I. *In vitro*, AS-IV (10 μM) significantly inhibited isoproterenol-induced proliferation of neonatal CF in rats. Its mechanism is related to the upregulation of miR-135a and, thus inhibition of TRPM7/TGF-β/Smads signaling pathway (Wei et al., 2020). Oxidative stress is closely associated with myocardial fibrosis. Dai et al. found that AS-IV (100 µM) inhibited isoproterenol-induced cardiac fibrosis by inhibiting ROS-mediated MAPK activation (Dai et al., 2017).

Previous studies have shown that inflammation plays an important role in the progression of cardiac fibrosis (Biernacka et al., 2011). The NLRP3 inflammasome plays an important role in the inflammatory response by promoting the maturation and secretion of various pro-inflammatory cytokines, including IL-18 and IL-1β (Abderrazak et al., 2015). Wan et al. found that AS-IV (200 mg/kg/day) reduced isoproterenol-induced cardiac fibrosis in BALB/c mice by inhibiting the NLRP3 inflammasome pathway (Wan et al., 2018). Pyroptosis is programmed cell death mediated mainly by caspase-1 and is accompanied by the release of many pro-inflammatory cytokines (Liu et al., 2016). Inhibition of NLRP3 inflammasome protects cardiac function and reduces myocardial pyroptosis (Takahashi, 2014). Gasdermins (GSDMs) are a key effector molecule involved in pyroptosis. Six human GSDM genes have been identified: GSDMA, GSDMB, GSDMC, GSDMD, GSDME, and DFNB59. The mechanism by which GSDMD induces the classical pathway of pyroptosis is quite clear. Zhang et al. found that AS-IV (40 mg/kg/day) alleviated myocardial fibrosis and hypertrophy in C57BL/6 J mice with AMI. Its mechanism was related to reduced pyroptosis by inhibiting the ROS/NLRP3/GSDMD signaling pathway. *In vitro*, AS-IV (100 µM) alleviated LPS-induced pyroptosis in bone marrow-derived macrophages (BMDMs) by inhibiting the ROS/NLRP3/GSDMD signaling pathway (Zhang et al., 2022a).

The intestinal flora and its metabolites play important roles in host heart health (Miele et al., 2015). Studies have shown that gut microbes are crucial in heart fibrosis (Karbach et al., 2016). Du et al. found that AS-IV (200 mg/kg/day) alleviated isoproterenol-induced cardiac fibrosis in C57BL-6J mice by increasing the richness of Akkermansia, Defluviitaleaceae_UCG-011, and Rikenella and regulating amino acid metabolism (Du et al., 2022). Ferroptosis is a recently discovered iron-dependent cell death pathway. Luo et al. found that AS-IV (10 mg/kg/d) inhibited ferroptosis by upregulating the Nrf2 signaling pathway, thus alleviating DOX-induced myocardial fibrosis (Luo et al., 2021a) (Figure 3).

### 2.8 Regulating autophagy

Autophagy is a lysosome-dependent pathway that degrades cytoplasmic material and damaged organelles (de la Ballina et al., 2020). Low levels of autophagy can phagocytize damaged organelles to maintain normal heart function; however, in chronic diseases such as diabetic cardiomyopathy, long-term autophagy may adversely affect cardiomyocytes (Guan et al., 2019). Under physiological conditions, B-cell lymphoma-2(Bcl2) protein binds to Beclin1; however, under starvation or oxidative stress, Bcl2 separates from Beclin1 and releases Beclin1 to induce autophagy. The mammalian target of the rapamycin (mTOR) signaling pathway is a classical pathway that regulates autophagy. The PI3K/Akt signaling pathway activates mTOR, thereby inhibiting autophagy (Kubli and Gustafsson, 2015). Zhu et al. found that AS-IV (100 µM) may inhibit HG-induced autophagy in H9C2 cells by regulating the miR-34a/Bcl2/(LC3II/LC3I) and pAkt/Bcl2 signaling pathways (Zhu et al., 2019a). Previous studies have shown that DOX induces myocardial injury by increasing cardiac autophagy (Luo et al., 2018). Luo et al. found that AS-IV (100 µM) inhibits autophagy and alleviates DOX damage in H9C2 cells by activating the PI3K/Akt/mTOR signaling pathway (Luo et al., 2021b). Myocardial I/R injury increases the production of reactive oxygen species (ROS), leading to autophagosome accumulation (Ma et al., 2012). Studies have shown that ischemia-induced autophagy is beneficial, whereas autophagy during reperfusion is destructive (Matsui et al., 2007). Huang et al. found that ASIV (50 μM) can protect myocardial I/R injury in C57BL/6 mice, and its mechanism is related to increasing superoxide dismutase (SOD) level to reduce the accumulation of superoxide radical O_2_^•-^ and thus reduce autophagy (Huang et al., 2021b) (Figure 3).

### 2.9 Improve microcirculation and promote angiogenesis

Microvascular obstruction independently predicts adverse cardiovascular events after AMI (Padro et al., 2020). Therefore, microvascular protection and promoting microvascular regeneration are important methods for reducing adverse cardiovascular events. Protein kinase D1 (PKD1), a member of the serine/threonine protein kinase family, promotes angiogenesis *in vitro* and *in vivo* (di Blasio et al., 2010). PKD1 may upregulate the expression of vascular endothelial growth factors (VEGF) by binding to the downstream target protein, histone deacetylase 5 (HDAC5) (Matthews et al., 2006). Yang et al. found that AS-IV (40 mg/kg/day) promoted angiogenesis in the myocardial tissue of AMI rats by upregulating the PKD1/HDAC5/VEGF signaling pathway (Yang et al., 2019). The signal transducer and activator of transcription 3 (STAT3) is a widely studied nuclear factor that plays an important role in angiogenesis in cardiac pathogenesis (Osugi et al., 2002). Sui et al. found that AS-IV (0.1, 0.3, and 1 mg/kg/day) promotes angiogenesis by upregulating the JAK/STAT3 pathway and alleviating HF in a left coronary artery ligation rat model (Sui et al., 2019). Sonic hedgehog (Shh) is required to maintain coronary blood vessels and is an angiogenic factor in ischemic diseases (Pola et al., 2001; Lavine et al., 2008). Wang et al. found that AS-IV (20 mg/kg/d) improved left ventricular remodeling and cardiac function in SD rats after myocardial infarction. Its mechanism was related to the upregulation of the Shh pathway to promote angiogenesis (Wang et al., 2017). The phosphatase and tensin homolog (PTEN) was originally thought to inhibit human cancer. However, PTEN affects the pathogenesis of cancer; for example, downregulation of PTEN can increase the expression of VEGF and promote angiogenesis (Jiang et al., 2000). Cheng et al. found that AS-IV (20 and 50 mg/kg/day) promoted the formation of capillaries in the boundary area of the infarcted myocardium in AMI rats. *In vitro* experiments, AS-IV (80 μmol/L) promoted the proliferation and formation of human umbilical vein endothelial cells (HUVECs). This mechanism is related to the inhibition of PTEN expression and activation of the PI3K/Akt signaling pathway (Cheng et al., 2019).

Reperfusion therapy for AMI is often accompanied by microvascular injury, manifested as microvascular leakage (MVL), which can aggravate tissue inflammation and edema and increase the occurrence of irreversible myocardial injury. The endothelial barrier is mainly regulated by junctions between adjacent endothelial cells, which are composed of transmembrane proteins, such as claudins, zonula occludens (ZO), occludins, vascular endothelial (VE)-cadherin and Catenins (Weis, 2008). He et al. (He et al., 2022) found that AS-IV (10 mg/kg/day) could reduce interstitial edema and inflammatory cell infiltration after myocardial I/R injury in SD rats and reduce coronary albumin leakage, the mechanism of which is related to the inhibition of the downregulation of junction proteins, such as Claudin-5, Occludin, ZO-1 and VE-cadherin. Previous studies have shown that ATP depletion can affect connexins and lead to the breakdown of the endothelial barrier. Previous studies have shown that downregulation of the insulin-like growth factor 1 receptor (IGF1R) signaling pathway can reduce ATP synthesis in the heart, and ATP depletion can affect connexins, leading to endothelial barrier breakdown (Li et al., 2014; Huang et al., 2019). *In vitro* studies, He et al. (He et al., 2022) found that AS-IV (10^–4^ M) increased ATP synthesis by upregulating the IGF1R signaling pathway in human cardiac microvascular endothelial cells (HCMECs), thereby preserving connexins and improving endothelial barrier function (Figure 3).

### 2.10 Antiviral myocarditis

Viral myocarditis (VM) is a major cause of HF and sudden death in healthy individuals. Many studies have shown that myocardial cell apoptosis plays an important role in the pathogenesis of VM. Liu et al. found that AS-IV (100 mg/kg/day) improved coxsackievirus B3 (CVB3)-induced myocardial cell apoptosis in mice, and its mechanism was related to the inhibition of FAS/FASL signaling pathways (Liu et al., 2019). A20, also known as TNF-α induced protein 3 (TNFAIP3), can inhibit CVB3-induced IKK complex activation and phosphorylation of IκBα. Gui et al. found that AS-IV (40 mg/kg/d) can inhibit NF-κB signaling pathway by increasing the expression of A20, reduce the severity of myocarditis induced by CVB3 in BALB/c rats, and reduce myocardial inflammation (Gui et al., 2015). Myocardial fibrosis plays an important role in CVB3-induced dilated cardiomyopathy (DCM). Chen et al. found that AS-IV could reduce myocardial fibrosis in DCM induced by CVB3 and improve ventricular dilation by down-regulating TGF-β1/Smad signaling pathway (Chen et al., 2011b). In addition, Zhang et al. found that AS-IV (60 and 120 mg/kg/d) can play an antiviral role in BALB/c mice infected with CVB3 by up-regulating the expression of interferon-γ (IFN-γ) mRNA (Zhang et al., 2006a) (Figure 3).

## 3 Vascular protection

### 3.1 Anti-vascular endothelial cell inflammation

Vascular endothelial cells (VECs) are present in the circulatory system and play an important role in maintaining vascular structure and function. The inflammatory response is a risk factor for endothelial dysfunction and an increased risk of cardiovascular events (Garcia et al., 2010). The NLRP3 inflammasome is a multiprotein pro-inflammatory complex that activates caspase-1 and cleaves pro-IL-1β and pro-IL-18 into mature IL-1β and IL-18 (Ozaki et al., 2015). Leng et al. found that AS-IV (40 and 80 mg/kg/d) reduced the activation of NLRP3 inflammasomes and the secretion of pro-inflammatory cytokines in the aorta of STZ-induced diabetic rats (Leng et al., 2019). In another study, Leng et al. found that AS-IV (100 μM) reduced the expression of intercellular cell adhesion molecule-1(ICAM-1), vascular cell adhesion molecule-1(VCAM-1), IL-6, and TNF-α in STZ-induced diabetic rat aortas by inhibiting the TLR4/NF-κB signaling pathway (Leng et al., 2018). Calpain is a calcium-activated cysteine protease located in the cytoplasm and mitochondria that plays a critical role in activating the NLRP3 inflammasome (Zhang et al., 2018a). Sun et al. found that AS-IV (50 and 100 μmol/L) inhibited the expression of NLRP3, caspase-1, IL-18, and IL-1βμ in human pulmonary artery endothelial cells (PAECs) induced by monocrotaline pyrrole (MCTP) through the NLRP3/calpain-1 pathway (Sun et al., 2021b).

**FIGURE 4:**
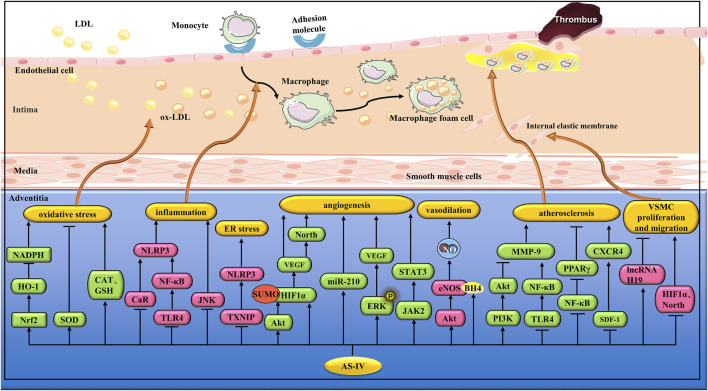
Pharmacological effects of AS-IV on blood vessels. AS-IV protects vascular endothelial cells by anti-inflammatory and antioxidant stress; AS-IV relaxes blood vessels, promotes angiogenesis, stabilizes atherosclerotic plaque, and inhibits vascular smooth muscle cell proliferation and migration.

Endoplasmic reticulum stress (ERS) is an adaptive response to unfolded protein accumulation and is associated with inflammation and apoptosis *via* the TXNIP/NLRP3 inflammasome pathway (Oslowski et al., 2012). Cycloastragenol is a microbial conversion of AS-IV, and both are present in the blood after the oral administration of astragalus (Zhou et al., 2012). Zhao et al. found that AS-IV combined with cycloastragenol reduced ERS induced by palmitate (PA) in EA.hy926 cells, and the mechanism was related to the inhibition of TXNIP/NLRP3 pathway activation (Zhao et al., 2015b). JNK is a serine/threonine protein kinase that is closely associated with cell differentiation, apoptosis, and stress response (Ip and Davis, 1998). You et al. found that AS-IV (50 μM) could reduce HG-induced apoptosis and inflammatory response in HUVECs by inhibiting JNK signaling pathway (You et al., 2019) (Figure 4).

### 3.2 Anti-oxidative stress of vascular endothelial cells

Endothelial dysfunction is associated with increased ROS production (Scioli et al., 2020). An imbalance between the production and elimination of intracellular oxides leads to the accumulation of intracellular ROS, which triggers oxidative stress. Zhu et al. found that AS-IV (10, 20, and 50 μM) enhanced the motility and migration of HUVECs induced by ox-LDL and inhibited ROS and NOX generation. This mechanism is related to the activation of the Nrf2/HO-1 signaling pathway (Zhu et al., 2019b). Hyperhomocysteinemia (HCY) is a risk factor for atherosclerosis (AS). Qiu et al. found that AS-IV (50 and 100 mg/mL) significantly improved HCY-induced HUVECs dysfunction caused by HCY by inhibiting ROS accumulation and increasing SOD activity (Qiu et al., 2010).

Studies have shown that chronic hyperglycemia can lead to endothelial dysfunction. Diabetic retinopathy is one of the main microvascular complications of diabetes, and hyperglycemia-induced oxidative stress plays an important role in diabetic retinopathy (Evans et al., 2002). Qiao et al. found that AS-IV (10 μM) significantly reduced HG-induced oxidative stress in retinal capillary endothelial cells (RCECs) by increasing SOD, catalase (CAT), and glutathione peroxidase (GSH-px) (Qiao et al., 2017). In another study, Nie et al. found that AS-IV (40 or 80 mg/kg/d) reduced ROS levels and increased SOD and GSH-Px activities in the aorta of diabetic rats (Nie et al., 2019b) (Figure 4).

### 3.3 Promote angiogenesis

Angiogenesis is an important pathological event in various chronic ischemic diseases, and remodeling angiogenesis around the ischemic area may be the most important strategy to improve the prognosis of ischemic diseases (Iwasaki et al., 2011). HIF-1α is a transcription factor that regulates the expression of several genes, including VEGF (Li et al., 2018a). Under normal oxygen levels, the HIF-1α protein is degraded by ubiquitination-mediated proteolysis, whereas under hypoxia conditions, HIF-1α preferentially undergoes SUMO modification, and the ubiquitination degradation pathway is inhibited (Wang et al., 2018). Wang et al. found that AS-IV (5 ng/mL) could stabilize HIF-1α protein by stimulating the production of SUMO1, thereby increasing the generation of VEGF and promoting angiogenesis under hypoxia conditions (Wang et al., 2021b). In another study, Zhang et al. found that AS-IV (0.25 μM) promoted tube formation in HUVECs and that its mechanism was related to the activation of the PI3K/Akt pathway, thereby promoting HIF-1α protein expression (Zhang et al., 2011). Liang et al. found that AS-IV (40 mg/kg/d) reduced cerebral infarction size and promoted angiogenesis in SD rats with middle cerebral artery occlusion. Its mechanism was related to the upregulation of miRNA-210 and the activation of the HIF/VEGF/Notch signaling pathway (Liang et al., 2020).

ERK1/2 play an important role in angiogenesis. Wang et al. found that AS-IV (10, 40, and 120 μM) could enhance the proliferation, migration, and tube formation of HUVECs by up-regulating the phosphorylation of ERK1/2 and activating the JAK2/STAT3 pathway (Wang et al., 2013). In another study, Wang et al. found that AS-IV (100 and 120 mg/L) upregulated VEGF synthesis by activating the ERK1/2 pathway, thus promoting angiogenesis in EA.hy926 cells (Wang et al., 2015). In addition, Zhang et al. found that AS-IV (10 μg/mL and 100 μg/mL) can promote tube formation of HUVECs by activating the Akt pathway (Zhang et al., 2012). Connexins (Cx) are a family of structurally dependent transmembrane proteins that form gap junctions. Among these, Cx37, Cx40, and Cx43 are closely associated with angiogenesis (Simon and McWhorter, 2003; Alonso et al., 2016; Leybaert et al., 2017). Li et al. found that AS-IV (0.2 μg/mL) can enhance the gap junctional intercellular communication function by up-regulating the expression of Cx37, Cx40, and Cx43, thus promoting the angiogenesis of endothelial cells (Li et al., 2018b) (Figure 4).

### 3.4 Vasodilation

Nitric oxide (NO) is a major endodermal relaxant that causes the relaxation of vascular smooth muscle cells (VSMCs) (Bian et al., 2008). Lin et al. found that AS-IV(10^–4^,10^–3^ and 10^–2^ M) dilates the rat aorta. *In vitro* studies, AS-IV increased the amount of NO in rat aorta endothelial cells (RAECs), which is related to the activation of the PI3K/Akt/eNOS pathway (Lin et al., 2018). Tetrahydrobiopterin (BH4) is a key cofactor in NO synthesis from eNOS. The absence of BH4 leads to the uncoupling of eNOS and the production of the superoxide radical O_2_^•-^ instead of NO (Landmesser et al., 2003). Xu et al. found that AS-IV (50 mg/kg/d) alleviated the vasoconstriction response induced by isoproterenol and inhibited the production of the superoxide radical O_2_^•-^ in the aorta of SD rats; the mechanism was related to the enhancement of BH4 levels and the inhibition of eNOS uncoupling (Xu et al., 2016a).

ROS can cause NO to form toxic peroxynitrite (OONO^−^), leading to the uncoupling of eNOS. Under normal physiological conditions, the NO produced by eNOS is sufficient to remove the superoxide-free radical O_2_^•-^ and protect cells from oxidative stress (Tesauro et al., 2006). However, when the level of the eNOS cofactor BH4 is not ideal, it leads to the uncoupling of eNOS, thus reducing the production of NO (Varadharaj et al., 2015). Xu et al. found that AS-IV (20, 50, 100 μmol/L) increased the generation of NO in HUVECs induced by hydrogen peroxide (H_2_O_2_), and the mechanism was related to the inhibition of the ROS/NF-κB pathway to reduce eNOS unconjugation (Xu et al., 2016b). In another study, Nie et al. found that AS-IV (40 or 80 mg/kg/day) increased NO production and eNOS expression in the thoracic aorta of diabetic rats by inhibiting calpain-1 (Nie et al., 2019b) (Figure 4).

### 3.5 Effects on atherosclerotic plaque

The destruction of atherosclerotic plaque stability is an important cause of cardiovascular and cerebrovascular diseases (Rosenfeld et al., 2000). Wang et al. found that AS-IV (40 mg/kg) reduced the lipid area of atherosclerotic plaques and increased collagen content and fiber cap thickness in ApoE^−/−^ mice fed a high-fat diet (HFD). Its mechanism is related to the regulation of PI3K/Akt and TLR4/NF-κB pathway, inhibition of MMP-9 expression, and anti-inflammatory. *In vitro* studies, AS-IV (5 mol/L) significantly reduced the ox-LDL-induced accumulation of cytoplasmic lipid droplets in RAW 264.7 macrophages induced by ox-LDL (Wang et al., 2020). In another study, AS-IV (10 mg/kg) reduced HFD-induced LDLR^−/−^ mice lipid levels, plaque area, and plaque stability by inhibiting the MAPK/NF-κB signaling pathway (Zhang et al., 2022b).

The formation and stability of atherosclerotic plaques are closely associated with inflammation. Studies have shown that peroxisome proliferator-activated receptor γ (PPAR-γ) is closely related to the production of inflammatory cytokines in macrophages (Oh et al., 2015). Sun et al. found that AS-IV (20 mg/kg/d) could upregulate PPAR-γ by inhibiting NF-κB, and reduce ox-LDL, TNF-α, IL-6, and IL-18 in the serum of SD rats, thus inhibiting the development of AS (Sun et al., 2018). OxLDL is also an AS promoter. In another study, Shao et al. established an AS cell model by exposing human umbilical HUVECs to ox-LDL. AS-IV (100 μM) reduced ox-LDL damage in HUVECs by regulating the circ_0000231/miR-135a-5p/CLIC4 pathway (Shao et al., 2021). Recent studies have shown that AS is associated with the stromal cell-derived factor-1 (SDF-1)/CXC chemokine receptor 4 (CXCR4) pathway. After platelet activation, SDF-1 is expressed in large quantities and binds to CXCR4, inducing the differentiation of CD34+ stem cells into macrophages and foam cells. Qin et al. found that AS-IV (40 mg/kg/day) reduced the degree of AS in the apoE^−/−^ mouse aorta by regulating the SDF-1/CXCR4 pathway (Qin et al., 2015) (Figure 4).

### 3.6 Anti-vascular smooth muscle cell proliferation and migration

Abnormal proliferation and migration of VSMCs are closely related to the occurrence and development of CVD, such as AS and restenosis. Platelet-derived growth factor (PDGF) stimulates VSMC proliferation and migration of VSMCs (Donovan et al., 2013). Chen et al. found that AS-IV (10 µM) could significantly weaken the effect of PDGF-BB on the proliferation of human dermal VSMCs (HDVSMCs) and inhibit the transformation of HDVSMCs from differentiation phenotype to proliferative phenotype. In addition, AS-IV can inhibit the expression of MMP2 protein and the migration of HDVSMCs by inhibiting the p38 MAPK signaling pathway (Chen et al., 2014). Cyclin-dependent kinases (CDKs) play a key role in the cell cycle. Among them, CDK2 can switch the cell cycle from G1 to S phase and regulate the G2 phase, thus promoting cell proliferation (De Boer et al., 2008). Zhang et al. found that AS-IV (10 µM) inhibited Ang II-induced proliferation of A10 cells (adult rat VSMCs) by decreasing CDK2 activity (Zhang et al., 2018b).

Pulmonary arterial hypertension (PAH) is a chronic progressive disease with high mortality rates. Inhibiting the inflammatory response of PAEC and reducing the abnormal proliferation of pulmonary artery smooth muscle cells (PASMC) are effective methods for PAH treatment. Jin et al. found that AS-IV (10 and 30 mg/kg/day) reduced MCT-induced pulmonary artery pressure increases and inhibited pulmonary artery remodeling in SD rats. *In vitro* studies, AS-IV (10–80 µM) inhibited hypoxia-induced proliferation and anti-apoptosis of human PASMCs and inhibited HIF-1α and p-ERK1/2 protein expression enhancement in human PASMCs (Jin et al., 2021). In another study, AS-IV (2 mg/kg) was shown to mitigate pulmonary artery remodeling in rats with PAH. *In vitro* studies, AS-IV (20 μmol/L) inhibited hypoxia-induced PASMC proliferation, and the mechanism was related to Notch signaling inhibition (Yao et al., 2021). Zhang et al. found that AS-IV (10 and 50 mg/kg/d) has a relaxing effect on the pulmonary artery of PAH model rats and reduces the serum levels of ET-1, Ang II, TNF-α, and IL-6 in PAH model rats. *In vitro* studies, AS-IV (50 μM) inhibited PASMC proliferation under hypoxic conditions, which is consistent with the conclusions of these two studies (Zhang et al., 2018c).

Mitochondria are the powerhouses of cells and are believed to be key regulators of cell death. Lu et al. found that AS-IV (50 μg/mL) reversed the decline in the production of ATP in Ang II-induced VSMCs. In addition, AS-IV prevented Ang II-induced ROS overproduction and increased SOD activity (Lu et al., 2015). In another study, Song et al. found that AS-IV (50 μg/mL) inhibited autophagy and mineralization of VSMC by up-regulating lncRNA H19 and inhibiting dual-specificity phosphatase 5 (DUSP5) (Song et al., 2019) (Figure 4).

## 4 Pharmacokinetics and toxicology of AS-IV

Most current reports on the pharmacokinetics and toxicology of AS-IV have been published in Chinese literature. Zhang et al. (Zhang et al., 2006b) found that more than 83% of AS-IV bound to plasma proteins, and the binding relationship with plasma proteins was linear in the concentration range of 250–1,000 ng/mL. The elimination half-lives of AS-IV (0.75, 1.5, and 3.0 mg/kg) were 98.1, 67.2, and 71.8 min in male SD rats, and 34.0, 66.9, and 131.6 min in female SD rats, respectively. The systemic clearance rate of AS-IV did not differ significantly, suggesting that it may have linear pharmacokinetic characteristics within the experimental dose range in rats. AS-IV also showed linear pharmacokinetic characteristics in beagles, with elimination half-lives of 51.9, 60.0, and 68.8 min in males and 62.9, 67.2, and 50.2 min in females at doses of 0.25, 0.5, and 1 mg/kg. The concentration of AS-IV was highest in the lungs and liver of rats, whereas its distribution in the brain was limited, possibly because of the difficulty of AS-IV penetrating the blood-brain barrier. In addition, only trace amounts of AS-IV were detected in the brain and testes of male rats (<0.10 μg/g), and lower levels were detected in the brain and ovaries of female rats (<0.26 μg/g). Zhang et al. (Zhang et al., 2006b) also studied the excretion of urine and feces in rats after intravenous administration of 1.5 mg/kg AS-IV and found that the total amounts of urine and feces excreted in male rats were 45.03% and 53.61%, respectively, indicating that nearly 50% of AS-IV could be metabolized *in vivo*. AS-IV is slowly cleared through the liver, with a systemic clearance (CL) of approximately 0.004 L/kg/min. In addition, the distribution of AS-IV in male and female dogs ranged from 0.23 to 0.36 L/kg (less than the overall level of 0.6 L/kg *in vivo*), suggesting that AS-IV may have limited distribution in the extravascular compartment.

Gu et al. (Gu et al., 2004) evaluated the transport and bioavailability of AS-IV. They found that the absolute bioavailability of AS-IV after oral administration was only 2.2% in a rat intestinal perfusion model. This low bioavailability may be attributable to its high molecular weight and poor solubility in water and lipids. Zhang et al. (Zhang et al., 2007) reported that the absolute bioavailability of AS-IV in beagle dogs was approximately 7.4%. The binding rate of AS-IV plasma protein was approximately 90% in the concentration range of 250–1,000 ng/mL. However, the low bioavailability of AS-IV limits its oral use.

Yu et al. (Yu et al., 2007) observed the subchronic toxicity of *Radix Astragali* extract (RAE) in SD rats and beagle dogs. The authors found that RAE was safe and did not cause significant toxic side effects. The safe dose range of astragaloside IV is 5.7–39.9 g/kg in SD rats and 2.85–19.95 g/kg in beagle dogs, which is 70 or 35 times of that in humans. Zhu et al. (Jiangbo et al., 2009) studied the effects of AS-IV on embryonic development in rats and New Zealand White rabbits. 0.25, 0.5, and 1.0 mg/kg/d of AS-IV were administered intravenously during 6–15 days of gestation in SD rats, and 0.5, 1.0, and 2.0 mg/kg/d of AS-IV were administered intravenously during 6–18 days of gestation in New Zealand White rabbits. The results showed that the fetal mortality of SD rats was significantly higher than that of the control group after the intravenous injection of 0.5 and 1.0 mg/kg/d. In a New Zealand White rabbit study, the stillbirth rate was significantly higher in each treatment group than in the control group. This suggests that AS-IV is fatally toxic at doses greater than 0.5 mg/kg/d. No major visceral abnormalities or skeletal malformations were observed in any group of rats or New Zealand White rabbits. Wan et al. (Xuying et al., 2010) evaluated the effects of AS-IV on perinatal reproductive toxicity in SD rats. They assessed rat fertility and early embryonic developmental toxicity by intravenous injections of 0.25, 0.5, and 1.0 mg/kg/d AS-IV 4 weeks before mating, throughout mating, and on day 6 of female gestation. They found no clinical signs of toxicity associated with AS-IV in male or female F0 rats before mating, during mating, or during pregnancy. No differences in the liver, kidney, or reproductive organs were observed. In addition, there were no significant changes in sperm count, percentage of motile sperm, or proportion of total abnormal sperm in male rats, but 1.0 mg/kg/d AS-IV reduced the mating index of rats. Next, we investigated the effects of AS-IV on physiological and reflex development in F1 rats. A maternal dose of 1.0 mg/kg/d of AS-IV resulted in significant delays in fur appearance, eye-opening, motor activity, and cliff avoidance reflex time. However, in the memory and learning tests, there were no significant differences between the two groups. As the expected clinical dose in humans is 10 mg/60 kg/day, which is comparable to the dose studied above, AS-IV should be used with caution in the treatment of cardiovascular and cerebrovascular diseases in perinatal women.

## 5 Discussion

In recent years, significant progress has been made in the research and development of therapeutic methods and drugs for CVD. However, CVD morbidity and mortality remain high, and the search for highly effective drugs with fewer adverse effects remains a priority. *Astragalus*, a commonly used Chinese herbal medicine, exerts protective effects on the cardiovascular system. Astragalus injection, in which AS-IV content is up to 11.30 mg/mL, has achieved good efficacy in the treatment of coronary heart disease, angina pectoris, viral myocarditis, and other CVDs. The protective pathway of AS-IV in CVDs is complex, and its protective mechanism is networked. For example, AS-IV (100 µM) inhibits isoproterenol-induced myocardial fibrosis by inhibiting ROS-mediated MAPK activation (Dai et al., 2017), suggesting that AS-IV improves myocardial fibrosis by inhibiting oxidative stress. AS-IV (200 mg/kg/day) alleviated isoproterenol-induced cardiac fibrosis in BALB/c mice by inhibiting the NLRP3 inflammasome pathway (Wan et al., 2018), indicating that AS-IV improves myocardial fibrosis by inhibiting inflammation. AS-IV (40 and 80 mg/kg/day) inhibited the degree of myocardial hypertrophy induced by AAC through upregulation of the Nrf2/HO-1 signaling pathway (Nie et al., 2019a), suggesting that AS-IV improved myocardial hypertrophy through antioxidants. Currently, studies on the pharmacological action and mechanism of AS-IV are not in-depth, and a regulatory relationship is lacking. Previous studies on the cardiovascular protective pathway of AS-IV may only be part of the downstream signaling pathway after the action of AS-IV on specific molecular targets. Therefore, in-depth studies of the specific molecular targets of AS-IV using systems biology approaches are urgently required. For example, a photoactive probe was used to label metformin, and PEN2 was identified as a direct target of metformin (Ma et al., 2022).

It should be noted that the results of different studies are not entirely consistent. Liu et al. (Liu et al., 2018) found that AS-IV (10 and 20 mg/kg/day) reduced myocardial hypertrophy, inflammatory response, and cardiomyocyte apoptosis induced by the aortic band in C57BL6 mice by inhibiting the TBK1/PI3K/Akt pathway. However, Wei et al. (Wei et al., 2019) found that AS-IV (5 and 10 mg/kg) alleviates myocardial I/R injury in SD rats by activating the PI3K/Akt signaling pathway. Zhao et al. (Zhao et al., 2015a) found that AS-IV (20 mg/kg) alleviates lipopolysaccharide (LPS)-induced cardiac dysfunction and inflammatory mediator production in male C57BL/6 mice by activating the PI3K/Akt signaling pathway. The regulation of AS-IV on PI3K/Akt has been inconsistent in different studies, which may be related to the accuracy of the experiments. GATA-4 regulates the survival and adaptive response of cardiomyocytes under stress conditions. Yang et al. (Yang et al., 2020) found that AS-IV (0.5–300 μg/mL) can promote the expression of Bcl-2 by stimulating the overexpression of GATA-4, thus reducing the apoptosis of H9c2 cells induced by H/R damage. However, Lu et al. (Lu et al., 2014) found that AS-IV reduces LPS-induced myocardial hypertrophy by inhibiting the expression of GATA-4. In these two studies, the regulation of the expression of GATA-4 by AS-IV was inconsistent, which might be related to the different intervention methods. HDAC are enzymes that regulate histone acetylation. Zhang et al. (Zhang et al., 2021) found that AS-IV (12.5 and 50 µM) could inhibit HDAC activity, thereby protecting HL-1 mouse cardiomyocytes from oxidative damage of ox-LDL. However, Yang et al. (Matthews et al., 2006) found that AS-IV (40 mg/kg/day) promoted angiogenesis in the myocardial tissue of AMI rats by upregulating the HDAC5/VEGF signaling pathway. In these two studies, the regulation of HDAC expression by AS-IV was inconsistent, which may be related to the target cell type. The Notch signaling pathway is also associated with angiogenesis. Liang et al. (Liang et al., 2020) found that AS-IV (40 mg/kg/day) promoted angiogenesis in SD rats with middle cerebral artery occlusion by activating the Notch signaling pathway. Yao et al. (Yao et al., 2021) found that AS-IV (20 mol/L) mitigated hypoxia-induced PASMC proliferation by inhibiting the Notch signaling pathway. In these two studies, AS-IV showed inconsistent regulation of the Notch signaling pathway, which may be related to the cell type or drug dose. In future studies, systems biology methods should be used to further study the specific molecular targets of AS-IV so that the mechanism of action of AS-IV can be fundamentally clarified.

## 6 Conclusion

In conclusion, AS-IV has multiple targets and pathways that play important roles in CVD. AS-IV protects the myocardium through anti-oxidative stress, anti-inflammatory effects, regulation of calcium homeostasis, improvement of myocardial energy metabolism, anti-apoptosis, anti-cardiomyocyte hypertrophy, anti-myocardial fibrosis, regulation of myocardial autophagy, and improvement of myocardial microcirculation. AS-IV protects VECs through anti-inflammatory and antioxidant stress pathways. In addition, AS-IV stabilized atherosclerotic plaques and inhibited VSMC proliferation and migration. AS-IV is a complementary or alternative medicine that cannot replace the primary therapeutic agent. In addition, most of the efficacy of AS-IV has been observed under laboratory conditions, and clinical studies have been relatively inadequate. Thus, AS-IV is an important drug worth exploring.

## Figures and Tables

**Table:** 

CVD	Cardiovascular diseases
I/R	Ischemia/reperfusion
ROS	reactive oxygen species
TCA	tricarboxylic acid
mPTP	mitochondrial permeability transition pore
GSK-3β	Glycogen synthase kinase 3β
DOX	Doxorubicin
NOX2	NADPH oxidase 2
HDAC	Histone deacetylases
ox-LDL	oxidized low-density lipoprotein
LPS	Lipopolysaccharide
TLR4	Toll-like receptor 4
TNF-α	tumor necrosis factor α
ATP	adenosine triphosphate
AMP	adenosine monophosphate
PPARα	proliferator-activated receptor α
CLP	cecal ligation and puncture
AMI	acute myocardial infarction
HMGB-1	high mobility group protein B1
MI	myocardial infarction
CaMKII	calcium/calmodulin-dependent protein kinase II
RyR2	ryanodine receptor 2
SERCA2a	sarcoplasmic reticulum Ca^2+^-ATPase2a
PGC-1α	Peroxisome proliferator-activated receptor-gamma coactivator-1α
H/R	hypoxia/reoxygenation
CaSR	Calcium-sensing receptor
cTnI	cardiac troponin
LDH	lactate dehydrogenase
CaN	Ca^2+^/Calcineurin
PLB	Phospholamban
SR	sarcoplasmic reticulum
HF	heart failure
TAC	transverse aortic contraction
SUMO1	small ubiquitin-like modifier1
FFA	free fatty acid
NRVM	neonatal rat ventricular myocytes
MCAD	medium-chain acyl-CoA dehydrogenase
MCPT-1	muscle carnitine palmitoyl transferase −1
NRF	nuclear respiratory factor
STZ	streptoxylotocin
MAPK	mitogen activated protein kinase
JNKs	c-Jun NH2-terminal kinases
HG/HF	high glucose and fat
HIF-1α	hypoxia-inducible factor-1α
RNCM	rat neonatal cardiac myocytes
SIKE	Suppressor of IKKϵ
TBK1	TANK-binding kinase 1
Ang II	angiotensin II
Nrf2	nuclear factor-erythroid 2-related factor 2
ECM	extracellular matrix
TRPM7	Transient receptor potential melastatin 7
α-SMA	α smooth muscle actin
CF	cardiac fibroblast
GSDMs	Gasdermins
BMDMs	bone marrow-derived macrophages
Bcl2	B-cell lymphoma-2
mTOR	mammalian target rapamycin
SOD	superoxide dismutase
PKD1	Protein kinase D1
VEGF	vascular endothelial growth factor
HDAC5	histone deacetylase 5
STAT3	Signal transducer and activator of transcription 3
Shh	Sonic hedgehog
PTEN	Phosphatase and tensin homolog
HUVECs	human umbilical vein endothelial cells
MVL	microvascular leakage
ZO	Zonulaoccludens
HCMECs	human cardiac microvascular endothelial cells
VM	Viral myocarditis
CVB3	coxsackie virus B3
TNFAIP3	TNF-α induced protein 3
DCM	dilated cardiomyopathy
IFN-γ	interferon-γ
VECs	vascular endothelial cells
ICAM-1	intercellular cell adhesion molecule-1
VCAM-1	vascular cell adhesion molecule-1
HPAECs	human pulmonary artery endothelial cells
MCTP	monocrotaline pyrrole
ERS	Endoplasmic reticulum stress
HCY	Hyperhomocysteinemia
RCECs	retinal capillary endothelial cells
Cx	Connexin
NO	Nitric oxide
RAECs	rat aorta endothelial cells
BH4	Tetrahydrobiopterin
H_2_O_2_	hydrogen peroxide
HFD	high fat diet
PPAR-γ	peroxisome proliferator activated receptor γ
SDF-1	stromal cell-derived factor-1
CXCR4	CXC chemokine receptor 4
CDKs	cyclin-dependent kinases
PAH	Pulmonary arterial hypertension
PASMC	pulmonary artery smooth muscle cells
DUSP5	dual-specificity phosphatase 5
